# The Relationship Between Dance Training Volume, Body Composition, and Habitual Diet in Female Collegiate Dancers: The Intercollegiate Artistic Athlete Research Assessment (TIAARA) Study

**DOI:** 10.3390/nu16213733

**Published:** 2024-10-31

**Authors:** Catherine Saenz, David J. Sanders, Samantha J. Brooks, Lacey Bracken, Aydan Jordan, Justen Stoner, Emaly Vatne, Marley Wahler, Ann F. Brown

**Affiliations:** 1 Exercise Science, Kinesiology, Department of Human Sciences, College of Education and Human Ecology, The Ohio State University, Columbus, OH 43210, USA; jjordan8@jacksonville.edu (A.J.); stoner.160@buckeyemail.osu.edu (J.S.); vatne.1@buckeyemail.osu.edu (E.V.); 2 Exercise Science, Department of Applied Physiology, Parkinson School of Health Sciences and Public Health, Loyola University Chicago, Chicago, IL 60660, USA; dsanders2@luc.edu (D.J.S.); marleyfwahler@gmail.com (M.W.); 3 Department of Movement Sciences, College of Education, Health & Human Sciences, University of Idaho, Moscow, ID 83844, USA; sbrooks.phd@gmail.com (S.J.B.); afbrown@uidaho.edu (A.F.B.); 4 Margaret Ritchie School of Family and Consumer Sciences, College of Agriculture & Life Sciences, University of Idaho, Moscow, ID 83844, USA; 5 Exercise Science, Brooks Rehabilitation College of Healthcare Sciences, Jacksonville University, Jacksonville, FL 32211, USA; ldennis3@ju.edu; 6 Human Performance Collaborative, The Ohio State University, Columbus, OH 43210, USA

**Keywords:** diet, health, muscle mass, body fat, low energy intake, dance, female athletes, artistic athletes, body composition

## Abstract

Background: This study’s purpose was to evaluate the relationship between dance training volume, body composition, and habitual diet in female collegiate dancers. Methods: Thirty-three female collegiate dancers from three dance programs (20.4 ± 1.05 yrs.; 165.4 ± 11.3 cm, BMI 21.3 ± 3.4 kg/m^2^) participated in “The Intercollegiate Artistic Athlete Research Assessment (TIAARA)” study. We assessed dance training volume, body composition, and habitual diet. Data were analyzed using descriptive statistics (means ± SD). Two-sample *t*-tests were conducted to compare reported intake values versus sports nutrition recommendations. Two-tailed Pearson correlations (r) were computed for body composition and dietary intake (*p* < 0.05). Results: Collegiate dancers were enrolled in 16 ± 2.09 semester credits, with 7.7 ± 3.8 credits as dance movement courses and an additional 3.0 ± 1.5 h/week of rehearsal time. Body composition assessments included fat mass (24.4 ± 6.8%), lean mass (LM) (42.4 ± 10.1 kg), and total body water (32.6 ± 4.6 L). Habitual diets reflected a low-calorie diet (1399 ± 648 kcal/d), with ~20% of dancers consuming a very low-calorie diet (≤1000 kcal/d). Dancers reported under-consuming dietary protein (54.3 ± 26.2 g) and carbohydrate (171.8 ± 77.8 g). LM was positively correlated with daily total energy (r = 0.333), fat (r = 0.37), protein (r = 0.349), and leucine intake (r = 0.352). Conclusions: Our findings emphasize the positive effect of adequate nutritional quantity and quality on body composition in female collegiate dancers.

## 1. Introduction

Collegiate dancers engage in artistic movements that are both physically demanding as well as aesthetically coordinated [1]. Successful dance performance requires muscular strength, power, and endurance [2]. Increasing skeletal muscle is foundational for all athletes, but this is especially true in collegiate dancers, where the sport is both the academic and performance focus. Training volume considerations traditionally include extended hours for technique classes, rehearsals, and performances, and for the collegiate dancer, this is on top of rigorous academic schedules and additional psycho-social stressors commonly found in the collegiate setting [3]. Ensuring the body can withstand these higher training volumes during the semester is imperative to reduce injury risk, and skeletal muscle mass becomes increasingly important to help manage physiological and biomechanical loads. Therefore, ensuring collegiate dancers have a healthy body composition, including higher levels of skeletal muscle mass proportions, is essential for both their sport performance and health [4].

Similar to collegiate athletes, collegiate dancers’ energy demands increase to account for semester stressors such as academic performance standards, poor sleep during the semester, and general social pressures of the collegiate setting [5]. Dietary considerations specific to sports demands [6] become vital to support energy levels, nutrient availability, and skeletal muscle mass [1]. Proper dietary approaches emphasize caloric quantity as well as quality, such as protein-rich dietary profiles, as these are foundational to sustaining performance and recovery during a dance semester [7].

Unique to collegiate dance, artistic athletes often experience added pressure to meet aesthetic standards and artistic performance expectations in addition to matching energy and nutrient needs [4,8,9]. This has led to a culture riddled with purposeful and inadvertent restrictive dieting practices [10]. Recent reports share that collegiate performing artists, including collegiate ballet dancers, fall below sports nutrition recommendations for macronutrients and select micronutrients [10,11]. Moore et al. completed a secondary analysis to examine female athlete triad risk amongst female collegiate ballet dancers using the cumulative risk assessment screening, previous medical records, and a 7-day dietary log [11]. This team found that 96% of collegiate dancers reported some level of nutrient deficiency (i.e., either low in a macronutrient or micronutrient compared to sports nutrition and general dietary recommendations), resulting in a high percentage of these athletes in a low energy availability state and a moderate triad risk. Torres-McGehee and researchers screened a wider net of female athletes, including performing artists, and found similar results of low energy availability with or without an eating disorder risk [10], suggesting that dancers may not have been purposefully reducing energy intake and may not have been aware of the downstream deleterious effects of negative energy balance. While these studies were able to assess low energy availability more pointedly, they were not able to conduct a more comprehensive dietary analysis relying on a focused seven-day dietary log. The combination of high exercise and physical demands matched with improper nutritional intake can lead to health, performance, and recovery impairments that weaken both acute and chronic dance development and negatively impact career longevity. In this light, more detailed and comprehensive dietary intake information will provide value to address these challenges and build targeted programs to tackle this issue.

Despite its importance, few studies have focused on how nutritional habits complement training and performance demands in artistic athletes, and fewer have connected dietary intake to healthy skeletal mass proportions, much less in the collegiate environment. College dance programs are increasing around the country [12], offering an opportunity to address these issues in a growing, and potentially vulnerable, athletic population. Additionally, the available works in collegiate dancers have been completed in singular program settings and have been plagued by lower sample sizes. While insightful data have been generated, it has been challenging to translate this information to a wider breadth of college dancers and dance programs since each dance department has distinct curricular specifics (i.e., training style emphases, curriculum and dance-credit load requirements, and student enrollments), which could add additional noise to the results and data outcomes. This highlights a clear gap in the literature and sets the foundation to further explore a more widespread assessment of collegiate dancer training volume and the relationship between body composition and nutrition by including more diverse dance programs and opportunities for higher research participation engagement. Results from a more comprehensive assessment would fill a critical gap in the literature related to how nutrition impacts tenets of the performance paradigm in collegiate dancers and provide insight into the current setting to identify areas of concern to further investigate and target [13].

Therefore, this study’s purpose was to evaluate the relationship between training volume, body composition, and habitual diet in female collegiate dancers from different college dance programs throughout the United States.

## 2. Materials and Methods

### 2.1. Participants

Thirty-six female collegiate dancers from three different university dance programs voluntarily participated in this cross-sectional, descriptive study entitled “The Intercollegiate Artistic Athlete Research Assessment (TIAARA)” study. TIAARA assessed dance training history and volume, body composition, and habitual diet. Collegiate dancers were recruited via classroom informational sessions, email, and word of mouth. All testing occurred mid-semester (either fall or spring), a few weeks before end of semester performance season began. At the time of testing, dancers were fully immersed in their dance courses and were participating in additional dance time to include auditions and/or performance piece rehearsals. Inclusion criteria included university students who declared dance as either their major or minor. Any students not enrolled as a dance major or minor or who were injured and not cleared to dance at the time of data collection were excluded. Interested collegiate dancers attended an information session after which they voluntarily signed an informed consent form developed, approved, and managed by the primary institutional review board and approved by all ethics committees at the participating institutions via a reliance agreement. Participation was completely voluntary and had no impact on their academic standing as a dance minor or major. Of note, the primary institution mailed all primary testing materials to the participating institutions (i.e., refractometer and bioelectrical impedance machine) to reduce variations that may arise from using different testing modalities and equipment pieces. Additionally, all institutions read from a similar script for testing instructions, completed all testing in the same order, and followed the same standard operating procedures for every testing measure to reduce variation and increase uniformity in the testing assessments.

### 2.2. Assessment Battery

**Figure 1:**
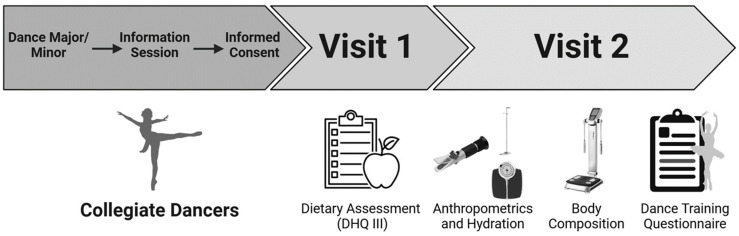
Experimental design. Visit 1 included a dietary assessment via the DHQ version III. For Visit 2, anthropometric and body composition were assessed followed by a vertical jump testing performed in both a traditional and dance-specific stance. Lastly, participants completed self-reported questionnaires that assessed dance history, academic and dance training schedules, and dance training volume.

Assessments were completed in two stages (Figure 1). First, dietary intake data were collected and analyzed using the Diet History Questionnaire (DHQ), version III, National Cancer Institute, Bethesda, MD (https://epi.grants.cancer.gov/dhq3, 26 January 2022). Following that, dancers attended their institution-specific human performance laboratory for the physiological and performance-based assessments between 0800–1030 h following an 8–10 h fast. Dancers were instructed to refrain from caffeine, alcohol, and strenuous exercise for 48 h before the laboratory visit but to consume water ad libitum in order to arrive euhydrated. Immediately upon arrival, dancers provided a urine sample for a urine specific gravity test (Atago 3749-E04, Tokyo, Japan) to assess hydration (set to <1.025). Any participant who did not arrive euhydrated (>1.025) was instructed to consume 8 oz of water and rest for 15 min before testing hydration again. If participants were unable to reach a euhydrated state within 30 min, their testing session was rescheduled.

### 2.3. Anthropometrics and Body Composition

Upon establishing a euhydrated state, participants completed a short adherence questionnaire and had their anthropometrics, such as height and weight, measured. Height (cm) was measured on a stadiometer three times and averaged to the nearest 0.01 cm. Body mass (kg) was collected on a medical-grade digital scale twice and averaged to the nearest 0.01 kg.

Body composition (fat mass (FM), lean mass (LM), and total body water (BW)) was assessed via bioelectrical impedance using the InBody 270 (InBody Cerritos, CA, USA) in a temperature-controlled room and following all manufacturer’s instructions.

### 2.4. Self-Reported Questionnaires

The final testing component was a self-reported questionnaire to capture dance training history and current training volume. Participants completed the survey on a cloud-based, password-protected platform with end-to-end encryption (Qualtrics, Provo, UT, USA). Dancers responded to questions related to topics such as what age they began dancing, enrolled credit hours during the semester, dance-specific credit hours during the semester, amount of time in various dance classes (i.e., ballet vs. jazz vs. contemporary dance weekly hour allotment), and time spent in either dance rehearsal or performing each semester. A list of the included questions can be found in Table 1.

### 2.5. Statistical Analysis

The results of this study were analyzed and reported using descriptive (means ± standard deviations (SDs)) and frequency statistics. Two-sample *t*-tests were conducted to compare reported values versus sports nutrition recommendations for dietary intake. Normality of differences was confirmed by visually inspecting normal Q–Q plots and Shapiro–Wilk normality test (*p* > 0.05). Two-tailed Pearson correlations (r) with Holm–Bonferroni correction were computed for body composition and dietary intake variables (*p* < 0.05). Finally, descriptive statistics of training volume were used to visually represent the weekly schedule of a collegiate dancer. All analyses were calculated using Microsoft^®^ Excel^®^ for Microsoft 365 Version 2403 and R Version (R Version 4.2.1 (R Core Team, Vienna, Austria).

## 3. Results

Of the thirty-six female collegiate dancers who volunteered to participate in this study, thirty-three completed the entire testing battery consisting of a training history and volume survey, body composition analysis, and dietary intake survey.

### 3.1. Dance Training History and Volume

Participants expressed starting dancing between the ages of 2 and 13 years of age and on average at 6.19 years old. Academically, participants were enrolled in an average of 16 total credits, of which 7.72 were dance training credits. Dancers reported additional rehearsal time occurring 3.0 ± 1.3 days/week for an additional 3.03 ± 1.5 h/week. Table 1 shows the mean and range results of the self-reported questionnaires.

### 3.2. Anthropometrics and Body Composition

**Figure 2:**
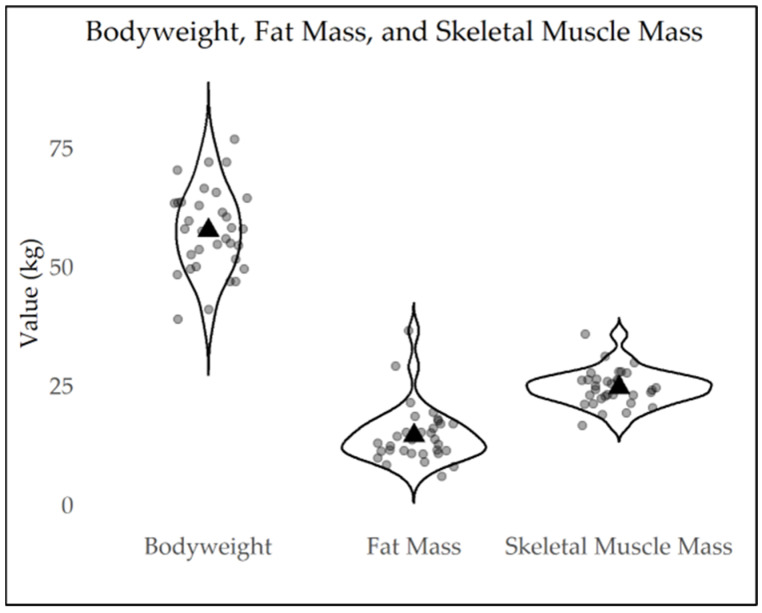
Distribution visualization of body weight, fat mass, and skeletal muscle mass of all participants.

On average, the dancers had a height of 165.46 cm ± 11.30 and a weight of 57.69 ± 8.91, resulting in a mean BMI of 21.34 kg/m^2^ (SD = 3.36). The participants’ body composition analysis showed a mean total body water of 32.60 L (SD = 4.55), fat mass (FM) of 14.46 kg ± 5.96 and 24.45% ± 6.77, lean mass (LM) of 42.41 kg ± 10.08 and 73.91% ± 14.52, and skeletal muscle mass (SMM) of 24.53 kg ± 3.75 and 42.64% ± 2.93. Anthropometrics and body composition results are presented in Table 2 as means and standard deviations (SDs), and a distribution visualization of body weight, fat mass, and skeletal muscle mass of all participants is presented in Figure 2.

### 3.3. Diet

**Figure 3:**
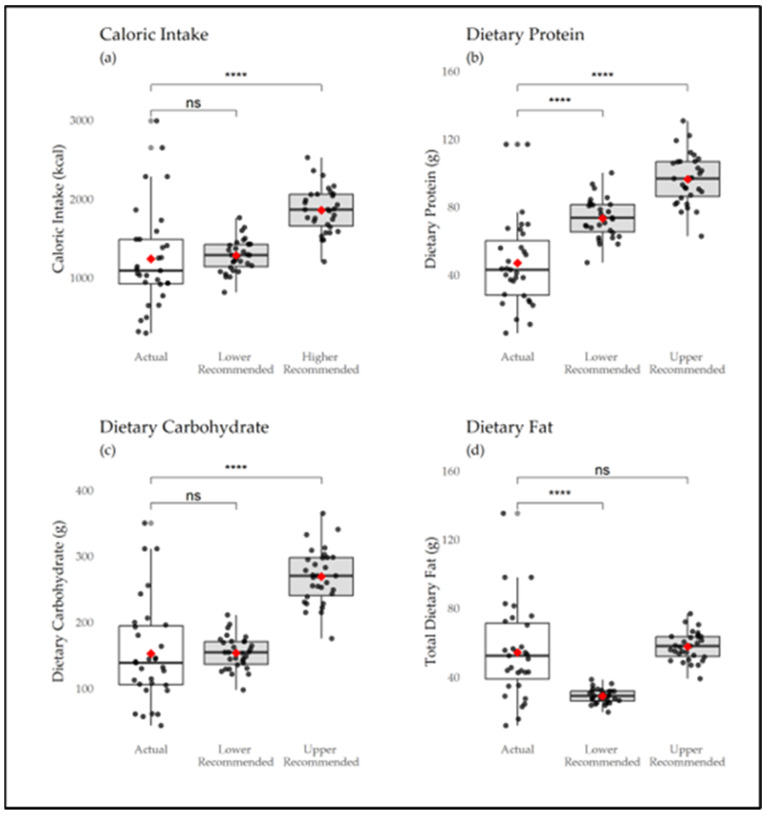
TIAARA-reported intake levels were significantly different for total energy (kcal), dietary carbohydrate (g), and dietary protein (g) but not dietary fat (g) when compared to current sports nutrition recommendation intake ranges. Each box plot showcases group means (red dot) and the individual intake level distributions. (**a**) Total energy; (**b**) protein; (**c**) carbohydrate; (**d**) total fat. “ns”, not significant; ****, *p* < 0.001.

Habitual diet reflected a low-calorie diet (1399 ± 647.87 kcal) with ~20% of dancers consuming a very-low-calorie diet of ≤ 1000 kcal daily, including two dancers consuming < 500 kcal/day. Dancers reported under-consuming dietary protein (54.3 ± 26.20 g) and carbohydrate (171.8 ± 77.8 g) but not dietary fat (54.19 ± 26.72 g) compared to sports nutrition recommendations. Two-sample *t*-tests demonstrated that dietary intake was significantly different from current sports nutrition recommendations [14] (Figure 3). Total energy intake was significantly less than the upper recommended value (*t* = −4.83, *p* < 0.01) but not the lower recommended value (*t* = −0.35, *p* = 73). Similarly, carbohydrate intake was significantly less than the upper recommended value of carbohydrate intake (*t* = −0.09, *p* = 0.93) but not the lower recommended value (*t* = −7.30, *p* < 0.01). Dietary protein intake was significantly lower than both the lower (*t* = −5.11, *p* < 0.01) and upper recommended values (*t* = −9.06, *p* < 0.01). Regarding specific amino acid intakes, participants reported mean values of isoleucine at 2.40 g (SD = 1.21), leucine at 4.17 g (SD = 2.09), lysine at 3.40 g (SD = 1.80), and methionine at 1.16 g (SD = 0.59). Table 3 shows the results of the dietary surveys, expressed as mean and standard deviation (SD).

### 3.4. Relationship Between Diet and Body Composition

Dietary profile, particularly total energy and total protein intake, was correlated with body composition. Greater reported energy was significantly associated with increased fat mass (*r* = 0.36, *p* = 0.048), lean mass (*r* = 0.43, *p* = 0.015), and skeletal muscle mass (*r* = 0.38, *p* = 0.034). Protein consumption was also significantly positively associated with fat mass (*r* = 0.36, *p* = 0.045), lean mass (*r* = 0.47, *p* = 0.008), and skeletal muscle mass (*r* = 0.41, *p* = 0.022). Increased dietary total fat was significantly associated with greater weight (*r* = 0.37, *p* = 0.041) and lean mass (*r* = 0.39, *p* = 0.031). Body composition variables (weight, fat mass, lean mass, and skeletal muscle mass) were significantly positively associated with one another. Similarly, all dietary intake variables were significantly positively associated. Table 4 shows two-tailed Pearson correlations (r) for body composition and dietary intake (*p* < 0.05).

## 4. Discussion

Collegiate dance programs include a unique subset of dancers that focus both on the physical and theoretical studies of dance science. Collegiate dancers undergo demanding dance and academic schedules yet have access to few resources, such as strength and conditioning programs or dietetic services, to support health and performance. To date, few studies have investigated nutritional habits in the context of a typical dance semester and its impact on body composition [4,13], and no studies have explored this question in a multi-site manner designed to capture a wider breadth of dancers and dance programs. This study was novel because it focused on the relationship between dance training volume, body composition, and habitual diet in female collegiate dancers in three distinct programs, a direction that addresses several gaps in the literature and sets a foundation for future intervention studies. The study’s primary findings were as follows: (1) Regardless of dance program, dancers reported consistent training volumes composed of a combination of dance credit hours and out-of-class dance demands such as rehearsals, additional training, and performances. (2) Compared to sports nutrition recommendations, collegiate dancers were under-consuming total calories, carbohydrate, and protein. (3) There was a positive relationship between LM and higher calorie, dietary protein, and branched chain amino acid intake.

Quantifying dance training demands provides a valuable needs analysis of what dancers require to physically prepare for each semester. Our study explored three distinct programs, and while specific dance classes differed between departments (i.e., ballet-focused vs. contemporary or theater-focused), dancers from all programs reported full credit semesters at 16 credit hours, with over half of these being accounted for by movement-based classes (7.72 h). The range of dance credit hours was quite large among respondents, with dancers reporting anywhere between 2–17 credits of movement-based coursework. This may be a reflection of a dance major compared to a dance minor; however, the majority of the participants were declared as dance majors. With such a large range in each dancer’s training volume, blanketed training suggestions to reduce issues such as overtraining fall short of personalizing the information to the individual dancer’s needs. Previous works have explored workload tracking through various wearables and sports science initiatives to bring insight to dance semester training intensity and volumes [2,15,16]. Sanders et al. reported a low training intensity in collegiate ballet and modern classes, which suggests a larger influence of volume (vs. intensity) on a dancer’s physiological demands [2]. Discussing training volume through the lens of credit versus non-credit dance time allows for a more translational metric that all collegiate dance programs may utilize as a workload metric. Future works could combine credit hour information with other workload measurements such as RPE and wearable technology.

From the responses observed in this study, it is evident that a higher dance volume leaves little room for attention to other foundational training needed to support performance, increase muscle mass, and reduce injury risk. Given the physical demands, an intuitive intervention should be to implement strength and conditioning sessions in the curriculum to elevate physical capacities [17]; however, currently, this is not a common addition in the college setting. The current schedule is completely focused on dance (Table 5), leaving little time to engage in any other type of strength-building activities. Increasing strength and conditioning opportunities has aided other collegiate sports by improving performance, reducing injuries, and better managing overall training loads [18]. Previous works in collegiate dancers have called to attention the need for increased strength training in this population; however, to date, few programs have added these foundational training programs into their curriculum [2]. College dance programs are unique in that classes contribute to workload, and rehearsal time is not managed by a governing body, such as is the case in varsity collegiate sports. This results in a wide range of sport-specific training volumes amongst collegiate artistic athletes. It is particularly important to understand dance training volume in collegiate dancers, as recent, though limited, works have warned this may be a population at risk for overtraining syndrome during later stages of the semester [19].

One of the more eye-opening outcomes of this study was the low- to very-low-calorie diets collegiate dancers were consuming despite engaging in a full semester’s worth of dance and academic classes, rehearsals, and an active performance schedule. While it has been previously reported that dancers, including collegiate dancers, tend to under-consume total calories and essential nutrients [20], to our knowledge, this is the first college study to identify caloric intakes this low and to explore *habitual* dietary profiles in collegiate dancers. To date, works have more commonly reported short-term 24-h, 3-day, or 7-day dietary assessments [13,15,20], which have offered important but small snapshots into the dietary habits of these artistic athletes. This broader time assessment using a validated food frequency survey captures dietary nuances that may be missed with shorter-term surveys and suggests these low-calorie ranges may be a chronic issue in collegiate dancers. Only one other study provided longitudinal diet data in collegiate dancers [13]. Researchers followed collegiate dancers over a two-year period, during which dancers completed a 3-day diet record and a body composition assessment twice a semester. Similar to our findings, dancers were under-consuming dietary carbohydrate, protein, and essential micronutrients. Our analysis found that of the 33 dancers who participated, nearly half of the athletes were in low caloric ranges, with 24% (8/33) of them consuming low-calorie diets (<1200 kcal) and another 18 (6/33) consuming very-low-calorie diets (<800 kcal), with 2 of these athletes consuming less than 500 kcal/day. Low- to very-low-caloric intake responses were observed at each site, suggesting a low-energy culture that may carry increased injury and health risks. These results mimic the larger dance population, as dancers are three times more likely to be at risk for abnormal eating behaviors and disordered eating tendencies [21]. Chronic low-energy intakes may lead to low energy availability and can eventually cascade into negatively systemic effects on health and performance termed Relative Energy Deficiency in Sports (REDs) [22]. While REDs has been studied in several different sports context, less information is available in dancers. A recent consensus identified a series of validated questionnaires on LEA and REDs that would translate well to dance [21].

Performance nutrition is a foundational component of the artistic athlete performance paradigm, and ensuring proper intake levels combined with optimal macronutrient profiles is essential to properly support both health and performance considerations in these athletes. Dance culture is plagued by complicated food relationships. Much dance research has explored the various tenets associated with disordered eating behaviors and attitudes and their impacts on dancer health and performance [23]. Most of this research has focused on adolescent groups [24] and professional companies [25], with less research available on how low energy intake, with or without disordered eating or abnormal eating behaviors, impacts collegiate dancers [21]. One obvious barrier is simply finding time in the schedule for meal breaks (Table 5). The current schedule provides minimal opportunity for sit-down meals and emphasizes the need for meal prepping and daily meal packing. This is particularly problematic for the collegiate dancer because they may not have access to cookware/environments; grocery stores; or the budget, knowledge, or skillset needed to prepare a full day’s worth of caloric and nutritionally healthy meals. Given the unique setting collegiate dancers perform under, such as the added academic and social stressors [3], these data emphasize the need for more research related to low energy intake, and possibly low energy availability and disordered eating behaviors, in this setting so that we can better contextualize the information to collegiate dancers. To the best of our knowledge, there are currently no research studies focused on potential food insecurity issues amongst collegiate dancers, which offers an excellent opportunity for future research. While education on the importance of nutrition is universally helpful, it would also benefit dance programs to identify ways to increase food options and food security amongst collegiate dancers.

In addition to low energy intake, dancers were also under-consuming carbohydrate and especially dietary protein compared to current sports nutrition recommendations specific to female athletes [26]. Dancers reported consuming ~1.06 g/kg protein, which falls well below the sports nutrition recommended intake range of 1.4–2.2 g/kg/day [26]. Interestingly, dancers reported consuming an average of 4.17 g/day of the branched-chain amino acid leucine, which fit within, albeit on the lower end of, the general recommendations of an average intake of 0.7–3.0 g/meal [27]. This suggests that dancers may be consuming higher-quality proteins but fall short of the total protein intake, a vital component of supporting anabolism and recovery needs. This likely results in a negative energy and nitrogen balance that increases time spent in a catabolic state. Over time, this would cause lower-than-ideal lean mass percentage in consideration of training volume. Indeed, Deutz et al. reported a higher %BF (and therefore lower relative lean mass) in athletes who spent more time per day in an energy deficit of >−300 kcals [28]. There is a distinct need to develop muscle mass in dance to maintain health and performance levels in this population. To further drive this message, our findings demonstrate a positive relationship between higher caloric, protein, and branch-chain amino acid intake and higher lean mass levels. This type of information can aid in providing positive messaging to collegiate dancers about the relationship between body composition, habitual diet, and the importance of food quantity and quality.

More recently, dance-specific literature has emphasized the importance of body composition assessments in this population. Body composition, with a focus on healthy muscle mass levels, is related to improved functional performance outcomes, such as increased strength, endurance, and power, and reduced recovery times and injury rates [29]. However, there is also a complicated history of the role of body positivity and correct messaging in the artistic athlete world [30]. Dancers have been expected to present with an “ideal” aesthetic that is comprised of a lean physique with low body fat levels [31], but this may also be associated with lower muscle mass levels as well [30]. Professional assessment of body composition provides a means to ensure healthy adaptation throughout one’s career, which avoids the limitations of relying on body mass index (BMI), a poor indicator of muscle and lean mass [32]. The current participants presented with a healthy BMI but with “poor” %BF [33]. This contradiction between BMI and %BF outcomes, coined as normal weight obesity [32], has been previously reported in college dance majors [20]. The current dancers were found to have a similar %BF to other college dance studies majors, despite different assessment techniques (bioelectrical impedance vs. air displacement) [2]. Even though the training volume for college dance majors is high (>18 h per week), their %BF is higher than other collegiate power-endurance athletes [34]. The discrepancy in body composition outcomes between college dance majors and college athletes suggests an opportunity to educate dancers on the importance of muscle mass for health and performance.

**Figure 4:**
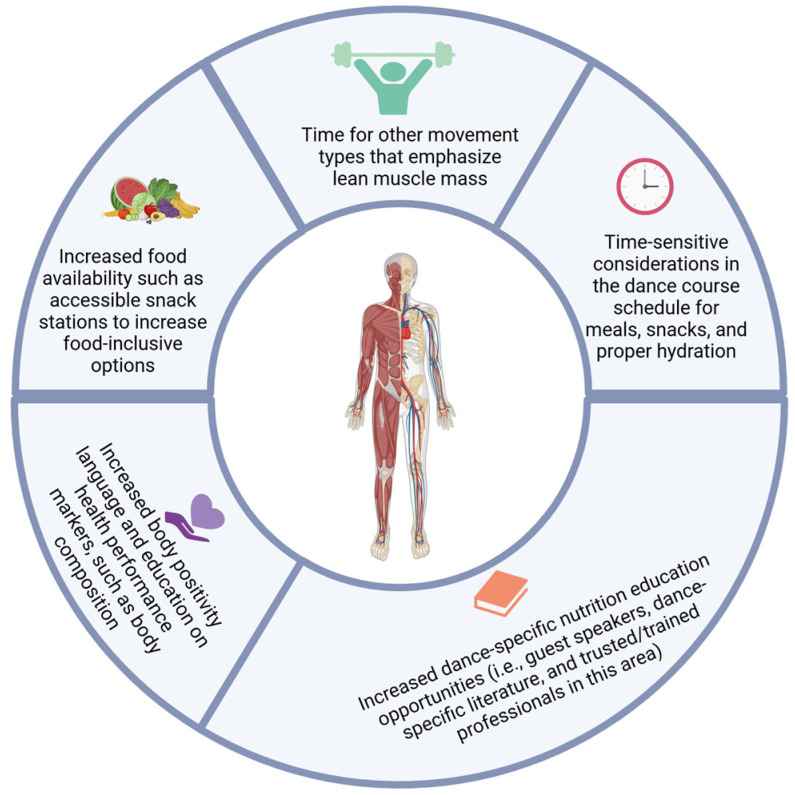
Actional recommendations collegiate dance programs can explore to support lean muscle mass.

One way to flip the narrative away from %BF, which can at times be more uncomfortable and less receptive, is to shift the focus to the importance of high-quality lean mass and the value of higher muscle mass percentage [29]. Anecdotally, dance programs often shy away from body composition measurements in part due to body image concerns and incorrectly digesting body composition results [30]. Indeed, these measurements should be collected by trained professionals who can properly educate on the value of body composition and appropriately analyze and review results with participants. However, body composition assessments should not be shied away from, as these data offer important health and performance-related information [29]. Therefore, while there are trends to limit its evaluation, an approach that emphasizes education and contextualizes results appropriately can have a positive impact on dancers’ health and performance outcomes. Notably, body composition results were shared in a conscientious manner with participants, with an emphasis on positivity and health. For example, body composition education was provided for any dancers who were interested. Before results were shared, body composition results excluded any information on group means or comparative values, and results were shared exclusively by the principal investigators to interested parties. Ensuring body composition is properly educated on is paramount to shifting the narrative to a more positive, muscle-centric, and health-focused approach. To help translate this information into practical application, dance programs are highly encouraged to explore sustainable nutrient and training options that support lean muscle mass in collegiate dancers. There are several lower-cost and assessable options that can be adopted (Figure 4). Additional research could explore how the implementation of these programs affects body composition, particularly lean muscle mass increases in collegiate dancers.

Although our study addresses several gaps in the dance science literature, this study also has several limitations that impact its translation to practical application. One of the study limitations was the general cross-sectional design. While much insight was gleaned from this snapshot approach, we were unable to capture how our primary outcomes, such as training volume, habitual diet, and academic stressors, may fluctuate throughout the semester and how various dance seasons may compare to each other. This was further challenged by the sample size, a common challenge in collegiate dance science research. Food frequency questionnaires have several limitations, such as underreporting, food bias, and vague questions that may be understood differently between respondents and may lead to ambiguous responses [35]. Considering the potential effects of underreporting energy intake, the low values observed would be marginally improved and likely yield similar conclusions. Additionally, our study focused on a single body composition assessment tool, bioelectrical impendence, and the two-compartment model approach. This method estimates fat-free mass and fat mass but is also particularly sensitive to external factors, such as hydration, that may impact the results. While our study controlled for hydration, there are inherent limitations to relying on any single body composition analysis as opposed to the emerging gold standard of a multi-compartment model approach (i.e., multiple body composition analyses combined to create four or more compartment models) [36]. Another limitation was the use of self-reported questionnaires to assess training volume and dance history, which relies on subjective data responses which can be prone to biases and inaccuracies.

There are several opportunities for future research to address limitations in our study and continued gaps in the literature. Future studies should explore multi-level diet reporting to capture diet data from several different angles, such as adding in more acute diet reporting and perhaps focus groups to capture dietary habits, behaviors, and schedules. While these techniques have been used individually in previous studies, to our knowledge, no studies have used them in combination. This information could capture more detailed information to better understand the low energy intake observed in this and other studies and begin to ask questions related to food security, food accessibility, and how the collegiate dancer’s daily schedule impacts food choices and consumption. Future works could center around more detailed performance nutrition assessments, intervention studies exploring various nutrition education programs, and nutritional support systems. Intervention studies could explore the use of dance-specific, registered dietician access; on-site meal planning support options; snack stations; increased body positivity language and education; and increased food/snack station options to explore how the presence of these lower cost, assessable programs impact dancer health, lean mass, and overall health and performance (Figure 4). They could also explore how factors beyond dietary protein impact lean muscle mass and body composition in collegiate dancers, such as micronutrient status, sleep, and academic stressors. Future studies could also adopt a multi-compartment model to help reduce noise and improve data validity [37]. Additionally, future dance studies, as well as dance programs in general, would greatly benefit from increasing body composition education. Better understanding of body composition and how skeletal muscle mass relates to health could aid in reducing stigmas and fears associated with body composition analyses often seen in the artistic athlete world. Future investigations would also be an excellent opportunity to explore more objective markers of energy expenditure, such as the addition of wearable technologies to provide more precise insight into the metabolic demands and exercise intensities amongst each dance program. These data could be combined with the perception of exertion during rehearsals and performances to provide more subjective data on the physical and psychological demands on dancers and contribute to better insight into dancer (i.e., player) load. These data could further inform the dance training demands collegiate artists are engaging in, identify opportunities for supportive training programs, provide insight to design more targeted intervention studies, and direct more individualized nutritional advice.

Lastly, a major area of future opportunity is expanding the participant pool in dance science. Dance science researchers and collegiate dance programs are often siloed in their unique university environments (various degree types and dance styles), making it challenging to collect data on a large sample size and report robust pilot data to achieve larger federal funding. A strength of our study was the ability to capture multi-site data and broaden our sample pool for a more comprehensive assessment of the collegiate dancer, yet it still was a smaller sample size than may be observed in larger athletic studies. In order to move dance medicine and science forward and understand collegiate dancers more holistically, numerous programs must be involved.

## 5. Conclusions

Collegiate dancers experience an increase in energy expenditure due to training and performances. We found that college dancers’ total caloric intake was insufficient to meet energy demands. Additionally, carbohydrate and especially dietary protein were below sports nutrition recommendations [14]. Further analyses demonstrated a positive relationship between skeletal muscle mass, total energy intake, total protein intake, and the essential amino acid leucine. These results call to attention the importance of addressing performance nutrition with collegiate dancers and the benefit of highlighting the essential nutrients needed for performance and recovery, such as total calories, dietary protein, and nutrient quality. They also emphasize the need for more specific analyses, such as targeted metabolic testing, objective training volume tracking, multi-compartment body composition analyses, and more comprehensive dietary assessments, to close the gap between food frequency questionnaires and much-needed energy expenditure assessments in the performing arts. It is vital to identify nutrition-related educational programs, resources, and food options that best fit the collegiate dancer’s needs and the barriers that may be impacting their use so that they can be incorporated appropriately into collegiate dance programs. Gaining a better understanding of what is impeding this population from adequate nutritional intake and improved body composition outcomes will help practitioners better develop individualized interventions to maximize dancer health and performance.

## Figures and Tables

**Table 1:** Dance training history and volume questionnaire.

Question	Mean	Range
At what age did you start dancing?	6.19	2–13
I am enrolled in ___ credits this semester.	16.0	12–21
I am enrolled in ___ dance classes this semester.	3.31	0–7
I am enrolled in ___ dance credits this semester.	7.72	2–17
I will be in ___ pieces this semester.	2.5	0–6
I will have rehearsal approximately ___ days per week this semester.	3.03	0–3
I will spend approximately _____ days per week at rehearsal past 8 pm.	0.77	0–3
Each piece will be performed approximately ___ time(s).	3.45	0–8
How many hours is your typical ballet class?	1.57	1–3
How many days per week do you take ballet?	3.33	0–6
How many hours is your typical contemporary/modern class?	1.65	0–6
How many days per week do you take contemporary/modern?	2.95	0–6
How many hours per week is your typical jazz, theatre, tap, or other dance-related classes?	0.67	0–3
How many days per week do you take jazz, theatre, tap, or other dance-related classes?	1.59	0–2

SD, standard deviation; Min, minimum; Max, maximum.

**Table 2:** Anthropometrics and body composition.

Question	Mean	SD
Height (cm)	165.46	11.30
Weight (kg)	57.69	8.91
Total Body Water (L)	32.60	4.55
BMI (kg/m^2^)	21.34	3.36
FM (kg)	14.46	5.96
FM (%)	24.45	6.77
LM (kg)	42.41	10.08
LM (%)	73.91	14.52
SMM (kg)	24.53	3.75
SMM (%)	42.64	2.93

SD, standard deviation; g, grams; kg, kilograms.

**Table 3:** TIAARA-reported intake levels compared to current sports nutrition recommendation intake ranges.

	Actual			Lower Recommended				Upper Recommended			
Diet	Mean	SD	Range	Mean	SD	*t*	*p*-Value	Mean	SD	*t*	*p*-Value
Total Energy (kcal)	1399.02	647.81	456.39–3149.46	1442.20	222.63	−0.35	0.73	2019.19	311.68	−4.83	<0.01 *
Protein (g)	54.30	26.20	12.99–124.28	80.76	12.47	−5.11	<0.01 *	103.84	16.03	−9.06	<0.01 *
Carbohydrate (g)	171.79	77.77	62.63–368.94	173.06	26.72	−0.09	0.93	44.53	44.53	−7.30	<0.01 *
Total Fat (g)	54.19	26.72	11.55–135.07	28.84	4.45	5.21	<0.01 *	57.69	8.91	−0.69	0.49

SD, standard deviation; Min, minimum; Max, maximum; *, significantly different when compared to current sports nutrition recommendations, *p* < 0.01.

**Table 4:** Two-tailed Pearson correlations (r) for body composition and dietary intake.

Variable	1	2	3	4	5	6	7	8	9	10	11	12
1. Weight (kgs)												
2. FM (kg)	0.79 **											
3. LM (kg)	0.53 **	0.40 *										
4. SMM (kg)	0.89 **	0.68 **	0.61 **									
5. Vertical Jump (cm)	0.18	0.30	0.24	0.15								
6. Energy (kcal)	0.34	0.36 *	0.43 *	0.38 *	0.23							
7. Protein (g)	0.33	0.36 *	0.47 **	0.41 *	0.23	0.95 **						
8. Total fat (g)	0.37 *	0.29	0.39 *	0.33	0.21	0.94 **	0.88 **					
9. Carbohydrate (g)	0.24	0.22	0.34	0.25	0.16	0.96 **	0.87 **	0.88 **				
10. Isoleucine (g)	0.34	0.41 *	0.49 **	0.44 *	0.25	0.94 **	0.99 **	0.84 **	0.83 **			
11. Leucine (g)	0.33	0.39 *	0.48 **	0.43 *	0.25	0.94 **	1 **	0.85 **	0.85 **	1 **		
12. Lysine (g)	0.31	0.40 *	0.49 **	0.44 *	0.25	0.90 **	0.98 **	0.78 **	0.79 **	0.99 **	0.99 **	
13. Methionine (g)	0.32	0.39 *	0.49 **	0.42 *	0.24	0.92 **	0.99 **	0.83 **	0.82 **	0.99 **	0.99 **	0.99 **

**, *p*-value < 0.01; *, *p*-value < 0.05; SD, standard deviation; kg, kilograms; g, grams; FM, fat mass; LM, lean mass; SMM, skeletal muscle mass; cm, centimeters; kcal, kilocalorie.

**Table 5:** Mean hours per week presented as a potential week schedule of a collegiate dancer.

Time of Day	Monday	Tuesday	Wednesday	Thursday	Friday
08:00 AM					
	Ballet (1.57 h)		Ballet (1.57 h)		Ballet (1.57 h)
09:00 AM					
	
10:00 AM					
	Contemp./Modern (1.65 h)	Academic Class #2 (1.5 h)	Contemp./Modern (1.65 h)	Academic Class #2 (1.5 H)	Contemp./Modern (1.65 h)
11:00 AM					
12:00 PM					
	Academic Class #1 (1 h)		Academic Class #1 (1 h)		Academic Class #1 (1 h)
13:00 PM					
				
14:00 PM					
	Rehearsal (3.03 h)	Other Dance-Related Class (1.47 h)	Rehearsal (3.03 h)		Rehearsal (3.03 h)
15:00 PM					

16:00 PM					
	
17:00 PM					
				
18:00 PM					
				

“h”, hours; contemp., contemporary.

## Data Availability

The original contributions presented in the study are included in the article; further inquiries can be directed to the corresponding author.
